# Evaluation of Titanium Dioxide Nanoparticle Suspensions as a Low-Cost Surface Coating to Improve Optical Profilometry of Transparent 3D-Printed Microdevices

**DOI:** 10.1021/acsaom.5c00010

**Published:** 2025-04-04

**Authors:** Ignatius Semmes, Gerard K. Lorio, Fannyuy V. Kewir, Jorge A. Belgodere, William Todd Monroe

**Affiliations:** 1 Department of Biological and Agricultural Engineering, Louisiana State University and Agricultural Center, Baton Rouge, Louisiana 70803, United States; 2 Tulane Department of Medicine, Section of Hematology & Medical Oncology, Tulane University Health Science Center, New Orleans, Louisiana 70112, United States; 3 Tulane Cancer Center, Tulane University, New Orleans, Louisiana 70112, United States

**Keywords:** titanium dioxide, Keyence VR-6000 Optical, surface coating, SLA printing, optical profilometry

## Abstract

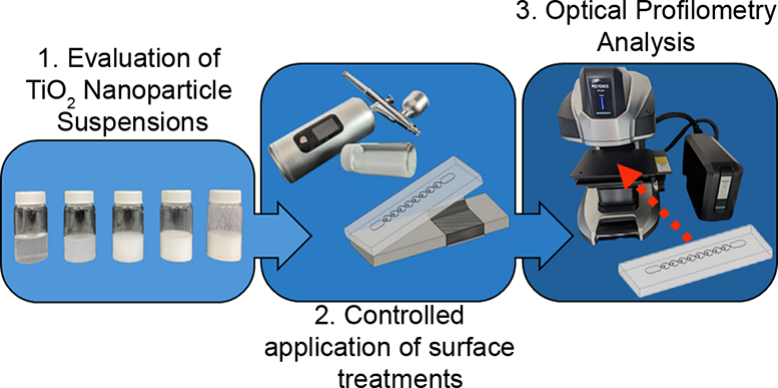

Improved resolution of stereolithography (SLA) 3D printers is accelerating the rapid prototyping of microdevices and has highlighted the need to evaluate their dimensional accuracy. Optical profilometry using structured light allows for rapid 3D scanning of devices with micrometer resolution but requires part surfaces with sufficient opacity and reflectivity for accurate measurement. Microfluidic devices are often made with transparent materials (e.g., clear SLA resins, PDMS, and glass), which poorly reflect the projected light, making them difficult to optically measure. To address the poor reflectivity of transparent objects, a low-cost titanium dioxide (TiO_2_) nanoparticle suspension was formulated to coat and opacify the surface of an object using a simple handheld airbrush. PDMS microdevices were cast from SLA printed molds to evaluate part geometry accuracy, surface roughness, and coating thickness between varying concentrations of the custom TiO_2_ spray, as well as commercially available 3D scanning sprays. TiO_2_ suspensions of 10 and 100 mg/mL in ethanol permitted accurate interrogation of parts of the features, yielding comparable results to commercial treatments. The performance of the treatments on different surface materials and channel designs was analyzed based on their intrinsic properties (roughness, thickness, and carrier solvent). The lower TiO_2_ concentration was preferable for microdevices with constricted features due to its lower coating thickness, while the higher concentration was favored for features with smaller z-heights due to its lower coating roughness, highlighting the need for tunable coating formulations. Cost, ease of use, and customization of the surface treatments were compared. The commercial treatments, in both the aerosol canister and microemulsion formats, were more time-effective due to minimal setup and cleaning requirements, whereas the custom TiO_2_ coatings were more cost-effective and customizable due to tunable properties and known composition.

## 1 Introduction

The advent of 3D printing has revolutionized the development of microdevices by enabling precise, customizable, and rapid fabrication of intricate structures at the microscale. These advancements have accelerated innovation and rapid prototyping in varying fields, from biotechnology to electronics. Stereolithography (SLA) 3D printing is an additive manufacturing technology that can construct complex models using photo-cross-linkable polymers, called resins, without additional tooling steps.^1^ Using a layer-by-layer approach, fabrication time is primarily dependent on the number of deposited layers, allowing for multiple replicates to be created with little penalty for overall fabrication time. The characteristics of SLA printing make it particularly suitable for producing low-volume, high-resolution microdevices inherent to microfluidics research.^2^ For example, a master mold can be designed, printed, and polydimethylsiloxane (PDMS) cast and cured to create a microfluidic device in ∼1–2 h.^3^

**Figure 1:**
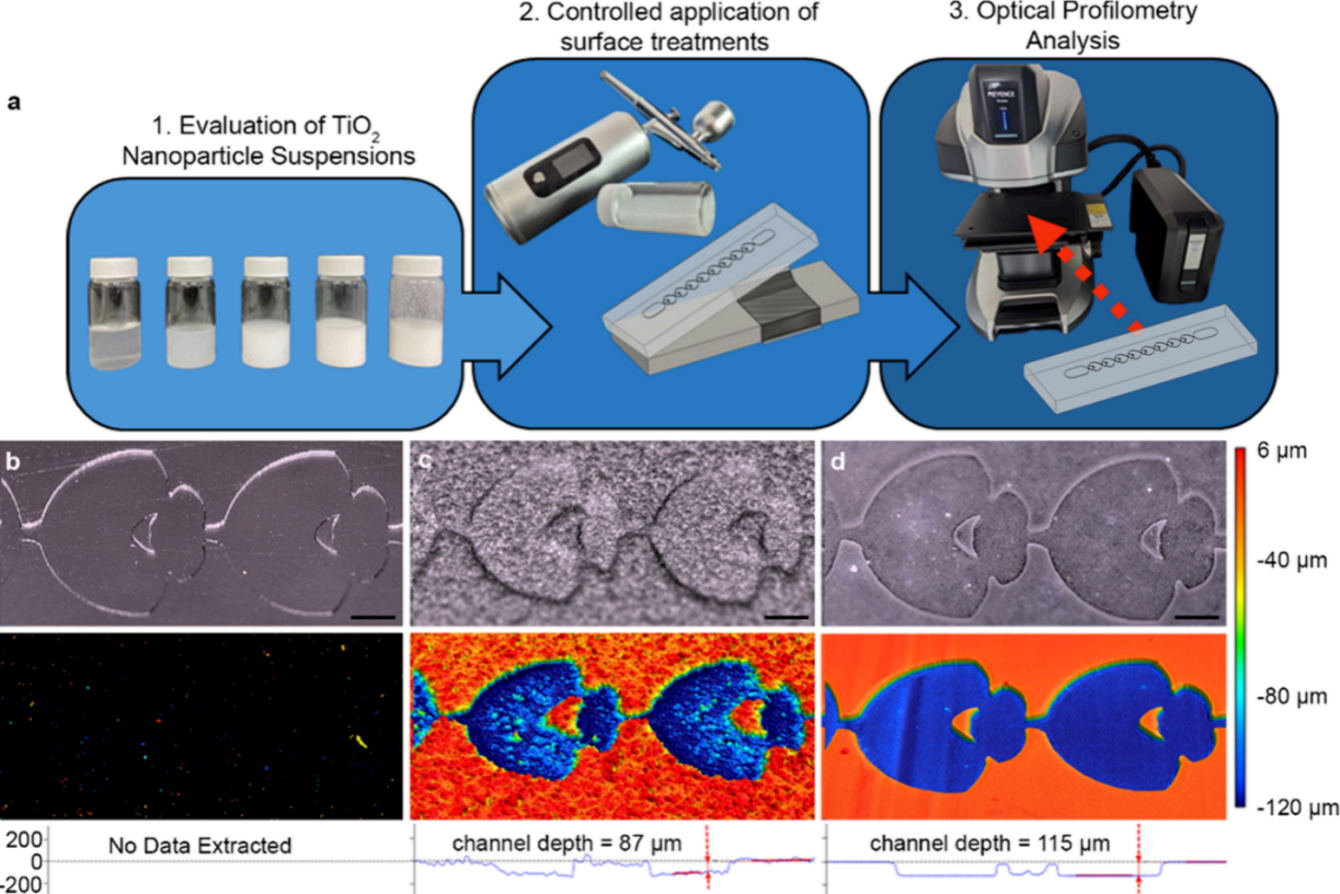
(a) Overview of the study, where TiO_2_ nanoparticle coating solutions were evaluated for improved optical scanning of 3D-printed microdevice prototypes. (b–d) Accurate optical profiling of transparent resin prints is challenging without surface treatment (b); unoptimized TiO_2_-based surface treatments (c) can obscure measurements of microchannel features, prompting the evaluation of customizable TiO_2_-based surface treatments for more accurate profiling of microstructures (d).

One drawback to SLA prototyping of microdevices at the resolution limits of these printers is the piece-to-piece variation, i.e., batch-to-batch variability, with feature dimensions differing from the intended design and also across prints.^4^ Dimensional differences become problematic when manufacturing microfluidic devices, where small alterations in geometry can affect device function. Thus, an iterative process of design, print, evaluate and redesign is often required. Optical profilometry provides a rapid and accurate method to evaluate print feature resolution and thereby characterize microstructure variation (Van der Jeught & Dirckx, 2016). Optical profilometers work by projecting light onto the part surface and detecting reflected light using sensors to develop a height mesh of the target object. The various methods of optical profilometry include laser triangulators, structured light profilometry (SLP), stereo vision, and photogrammetry.^5^ SLP is an example of an inexpensive profilometry technique that is operationally straightforward and can quickly create precise surface measurements. SLP is distinct in that a moving pattern of light is projected onto the target surface for a high-resolution overhead camera sensor to capture.^6^ Problems arise when optically scanning transparent surfaces that allow for all light to pass through or highly reflective surfaces, which reflect all light in a singular direction via specular reflection. As many microfluidic devices are specifically designed to be transparent for microscopic interrogation, reflected light is minimal, making optical scanning ineffective without some modification to the process (Figure 1b).

Solutions that facilitate the profiling of reflective and transparent materials have focused on either the development and utilization of specialized 3D scanning techniques or the application of nondisruptive coatings to part surfaces. Some examples of modified 3D scanning techniques include the ray propagation model, the high dynamic fringe acquisition method, optical projection tomography, UV irradiation, and the multiviewpoint 3D inspection system.^7−10^ Because the complexity of these approaches has limited their adoption in commercial optical profiling instruments, the surface coating method is often preferred. The surface coating method involves deposition of a thin layer of coating, in most cases composed of a nanoparticle mixture, onto a measuring object, which enables an optical profiler to accurately scan transparent and ultra reflective materials. However, an inherent disadvantage of this method is that high coating thickness and low uniformity on the microscale could greatly impact measurement accuracy (Figure 1c). Thus, highly tuned coating solutions are sought for the accurate scanning of parts with micrometer-scaled features. Surface coating solutions are commercially available in an aerosol spray form or as a liquid solution, the latter requiring its own application apparatus such as an air brush or atomizer.^11,12^ These prepackaged solutions lack tunability and characterization, which could limit their effectiveness when analyzing a wide variety of microfluidic materials and device resolutions. Customized nanoparticle-based alternatives can be developed as liquid solutions, which are applied via spraying, or as dry nanoparticles (e.g., gold, carbon, platinum, and silver). The application of dry nanoparticles requires expensive instruments such as sputter coaters for application onto a measuring object’s surface.^13^

While the sputter coating technique can produce highly effective thin and uniform surface coatings, their removal can cause permanent modification to part surfaces because of the requirement to use strong solvents.^13^ A less expensive alternative, atomization, applies a thin and uniform coating via internal ultrasonication and the propulsion of a nanoparticle solution onto the surface of a measuring object. However, atomizers require fine-tuning of the device for best results.^12^ An air brush is a relatively inexpensive and easy to use coating application device available in a variety of form factors. Specifically, some commercial airbrushes have on-device battery-powered compressors, which remove the bulk of traditional airbrush equipment for simplified operation. Airbrush application of thin coatings to dull highly reflective parts has permitted high-resolution optical scanning.^14^ Solutions of nanoscale titanium dioxide (TiO_2_) powder in ethanol have been demonstrated to effectively create coatings comparable to other custom or commercial sprays.^15^ Compared to the solvents used in commercially available aerosol canister sprays or the subliming coating sprays, ethanol would be preferred for its relative safety and green chemistry classification.^16^ Similarly, the U.S. Food and Drug Administration’s listing of TiO_2_ as Generally Regarded as Safe (GRAS) status^17^ is promising when working with biological systems, an important factor when working with microfluidic devices.^18^ The high light scattering, high refractive index, and relatively small agglomeration size of TiO_2_ nanoparticles that have promoted their wide use in paints, topical sunscreens, and cosmetics also make them ideal for optical profilometry.^19^ While commercial applications of TiO_2_ coatings have grown, there has been less reporting on their use in the optical scanning of transparent objects to measure feature dimensions at the microscale. Most work regarding surface coatings has focused on flat, reflective objects and not transparent parts with small, recessed features that could be obscured with nonoptimized coatings. With the increasing role that 3D printing and other rapid prototyping methods have in the creation of PDMS and other transparent microfluidic devices, accessible methods of microfeature analysis on these devices are becoming increasingly important in rapid prototyping processes.

The goal of this study was to develop an inexpensive, rapid, and consistent surface coating method to enable feature profiling of clear microfluidic devices, both resin 3D-printed and PDMS-casted. The specific objectives of the study were to (1) prepare and characterize TiO_2_ suspensions in ethanol and water; (2) optimize concentration and spraying methods of TiO_2_ surface spray solutions on microdevice parts, considering both surface roughness and accuracy of feature depth measurements; and (3) compare solutions to commercially available coating sprays. Development of these solutions and spraying methods would provide a customizable, inexpensive, green chemistry approach for the application of a thin, consistent surface coating on any reflective or transparent surface.

## 2 Materials and Methods

### 2.1 Preparation of TiO_2_ Solutions

Surface spray solutions were created, 0.01, 0.1, 1, 10, and 100 mg/mL, by dissolving XF-NANO TiO_2_ nanoparticle powder (20–40 nm stated diameter, Jiangsu Xfnano Materials Tech Co., Nanjing, CN) in ethanol (200 proof, Lab Alley, Austin, Texas, USA). Nanoparticles were weighed in glass scintillation vials (LanJing, Jiangsu, CN), 10 mL of solvent was added, and solutions were vortexed using a Vortex Genie 2 vortex mixer (Scientific Industries, New York, USA) for 1 min. Prior to use, solutions were vortexed to ensure a homogeneous solution.

### 2.2 Dynamic Light Scattering (DLS) and Scanning Electron Microscopy (SEM) Characterization of TiO_2_

For dynamic light scattering (DLS) analysis, TiO_2_ nanoparticles were resuspended in DI or ethanol at 1 mg/mL, sonicated at 100 W (Ultrasonic cleaner, BRANSONIC, USA), and filtered through a 0.22 μm filter (Argos Technologies, Illinois, USA), and the size was measured by a Zetasizer Nano ZS (Malvern Instruments, Malvern, UK). For scanning electron microscopy characterization, TiO_2_ nanoparticle solutions (100 and 10 mg/mL) and a commercially available spray (Hellings 3D) were applied onto SEM sample holders and imaged using a JSM-6700F (JEOL Ltd., Tokyo, Japan) at 5 kV, 6.3 pA, with a working distance of ∼3.3 mm, and imaged at 5000× and 40,000× magnifications.

### 2.3 Design and 3D Printing of Microfluidic Molds

To compare surface coating treatments, a microfluidic device mold was designed in Fusion360 (Autodesk, California, USA), adapted from a passivated unidirectional valve design.^20^ The design was chosen because of its incorporation of both raised and recessed features, varied channel widths, and complex design (Figure 1). Such geometries facilitate the accurate evaluation of the surface coating success across a wide range of potential microfluidic designs. Channel height was set to 80 μm, and outer walls of 1 mm were created around the design to facilitate casting. The design was imported into Lychee slicer software (version 6.0.200, Mango 3D) as an.STL file, sliced using optimized settings, Table 1, and 3D-printed using a Sonic Mini 8k 3D resin printer (Phrozen, Washington, DC. USA) with Aqua 8k gray resin (Phrozen, Washington, DC. USA). After printing, the microfluidic mold was postprocessed by ultrasonication in an IPA solution for 3 min and dry ultraviolet light curing for 10 min. To prepare the mold for casting, the mold was clamped between two standard glass microscope slides (VWR, Pennsylvania, USA) and heat cured at 75 °C for 30 min to remove any resin leachates. Once cooled, the mold was ready for casting.

**Table 1:** 3D Printer Settings

burn in layers		normal layers	
number of layers	4	layer height	10 μm
exposure time	50 s	exposure time	0.8 s
lift distance	4 mm	lift distance	5 mm
lift speed	60 mm/min	lift speed	60 mm/min
retraction speed	100 mm/min	retraction speed	100 mm/min

A 10:1 mixture (elastomer:curing agent) of Sylcap 284-F polydimethylsiloxane (PDMS, MicroLubrol, New Jersey, USA) was cast onto the molds, degassed in a vacuum chamber, and cured in an oven at 65 °C for at least 40 min. PDMS was demolded and cleaned with IPA and DI water, followed by drying with nitrogen. To create an opaque control cast that could be accurately scanned in the optical profiler and used as a control for comparison to the sprayed casts, iron oxide powder (FeO), Color Rare, Bordeaux, FR) was mixed in with the PDMS at 1% concentration by weight.

### 2.4 Surface Coating of Custom TiO_2_ Solutions and Commercial Sprays

Prior to applying the surface coating, the custom and commercial solutions were mixed or shaken following the manufacturer’s instructions. For custom TiO_2_, prepared solutions were vortexed and sonicated (1 min). To apply surface coatings, an airbrush was used. When applicable, 1 mL of the solution was deposited into the reservoir of a portable cordless airbrush (MGGSUG, Shenzhen, CN) immediately prior to application. Surface coating solutions in aerosol canisters were applied following the manufacturer’s instructions. Spraying was performed in a sweeping motion, 6 in. from the target pieces, which were suspended above the benchtop. A total of five spray applications were performed perpendicular to the piece for best coverage,^21^ with profilometry measurements taken after each layer application. In this way, each application represents one layer of the coating. This protocol was conducted in triplicate for each coating solution.

Profilometric scans were conducted within the 10 min of initial application to avoid any external interference with the coatings, as well as to avoid any deterioration of the sublimating sprays investigated here. Each surface coating solution tested in this study is described in Table 2.

**Table 2:** Description of Commercially Available Surface Treatments and the Custom Solution Studied Herein

**surface treatment**	**classification**	**application method**	**composition**	**price per unit volume^a^**	**source**
nanoparticulate TiO_2_	air brush application of solid particle suspension in solvent (AB-SPS)	air brush	TiO_2_ nanoparticle powder suspended in 100% ethanol	(10 AB-SPS) ∼$20.00/400 mL	this study
				(100 AB-SPS) ∼$42.00/400 mL	
Hellings Spray	aerosol canister spray application of solid particle suspension in solvent (AC-SPS)	aerosol Can	TiO_2_ nanoparticles suspended in Proprietary carrier solvent	$30.00/400 mL	Helling GmbH
Aesub Orange	aerosol canister spray application of microemulsion (AC-ME)	aerosol Can	proprietary sublimating microemulsion	$42.00/400 mL	Scanning-spray Ver-triebs GmbH
Aesub Yellow	air brush spray application of microemulsion (AB-ME)	air brush	proprietary sublimating microemulsion	$58.00/200 mL ∼$116.00/400 mL	Scanning-spray Ver-triebs GmbH

a The price of the AB treatments does not include the upfront costs of the air brush.

### 2.5 Profilometry and Analysis of Feature Depth Accuracy and Comparative Surface Coating Roughness

Test pieces were optically scanned by the VR-6000 series 3D optical profilometer (Keyence, Illinois, USA) after each coating layer was applied. The profiler’s detection camera was focused on an identical reference surface at 160× digital magnification before each scan. All scans were conducted at the maximum 40× optical magnification setting of the profiler using “Fine” and “High-Resolution (roughness)” detection settings in the VR-6000 Series Viewer Software (Version 4.2.0.457) (Keyence, Illinois, USA).

Profilometric scans were evaluated using VR-6000 Series Analyzer Software (Version 4.2.2.1116) (Keyence, Illinois, USA). Line profiles were collected using the vertical line tool perpendicularly to the microfluidic channel, revealing the channel’s recessed cross-section. The average feature depth of the channel cross-section was calculated with the “Line-to-Line” tool. To evaluate surface roughness of spray coatings, three measurement areas were sampled and the arithmetical mean height (Sa) was calculated. This method calculates the average absolute value height difference of each point of the optical scan compared to the arithmetical mean of the surface (Keyence, 2024). All scans were taken in the same area, and the analysis tools described above were deployed in a batchwise fashion.

A heatmap of an index combining measured part geometry accuracy and coating surface roughness was generated using GraphPad PrismTM to better visualize how each surface treatment and coating number affected feature resolution and evenness. Each box represents the value of the “Coating Index”, calculated by one-half of the sum of the average of the ratio of mean feature depth of the sprayed part to that of the opaque control and the average ratio of the surface roughness (Sa) of the sprayed part to the opaque control, averaged over the *n* = 3 replicates, as shown in eq 1.1:
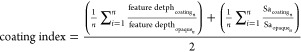
1.1

### 2.6 Analysis of Coating Thickness and Intrinsic Surface Roughness

The intrinsic surface roughness created by each coating was determined by applying a coating of surface treatment to a clean and polished silicon wafer substrate. Because the roughness of the silicon wafer is less than the optical profilometer’s detection range of approximately 1 μm, this facilitated the unobstructed analysis of the surface treatments. Following the application of each surface treatment, the intrinsic surface roughness was found by using the same approach as described previously. Three areas’ Sa measurements were averaged to find the approximate surface roughness of the entire coated surface. To determine the coating thickness of each surface treatment, a gray 3D-printed block was partially masked with labeling tape (VWR, Pennsylvania, USA), sprayed, unmasked, and then profiled across the step interface. The pigmentation of the gray-colored resin printed piece was optically opaque enough to profile without spray coating. The coating thickness was measured by comparing the average measurement heights across two sample areas (with and without nanoparticle coatings).

### 2.7 Statistical Analysis

Statistical analyses were performed using GraphPad Prism (v10.0.2, GraphPad Software, San Diego, CA). One- or two-way ANOVA with multiple comparisons (Dunnet or Tukey) were performed to determine significance of recorded values indicated in the figure legend. *p*-Values of <0.05 were considered significant. Each value is presented as the mean ± standard error of the mean (SEM). Any values exceeding 3σ from the mean were identified as outliers and removed from the data set. Further, some profiled results were unable to be measured and thus had no corresponding values included in the data set.

## 3 Results and Discussion

### 3.1 TiO_2_ Solution Preparation and Characterization

**Figure 2:**
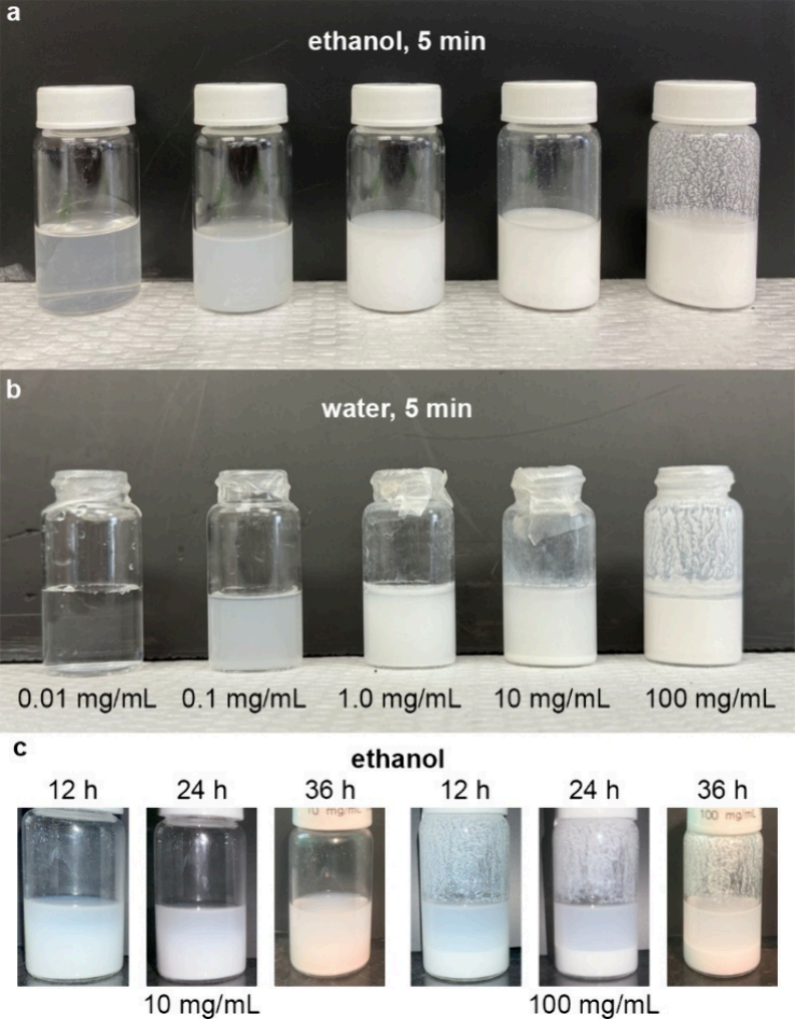
Varying concentrations of TiO_2_ nanoparticles in (a) ethanol and (b) water, photographed 5 min following initial preparation and mixing. Sedimentation at the higher concentrations was observed over hours (c), thus prompting the method of vortexing immediately prior to use.

To maximize the coating effectiveness of the TiO_2_ nanoparticle solutions, solubility was evaluated in DI water and ethanol. DI water and ethanol are commonly used within most laboratories and were selected for this study due to their green chemistry classification and similar use in scanning highly reflective metallic surfaces.^21^ Concentrations of the TiO_2_ nanoparticle suspensions (0.01–100 mg/mL) were prepared in either solvent and observed for sedimentation 5 min after preparation. The suspensions prepared in ethanol showed no signs of sedimentation, except at the highest concentration (100 mg/mL, Figure 2a). Similarly, the water solution exhibited sedimentation only at higher concentrations (10 and 100 mg/mL, Figure 2b). A previously reported study noted sedimentation at higher concentrations of TiO_2_, with degree of sedimentation varying across the sources of the nanomaterial.^21^ Further, they noted that for aggregates that fell out of solution spraying with an air brush required additional cleaning. This was also observed to a minor degree when spraying with the 100 mg/mL concentration of the TiO_2_ solution in this study. A longer time course of sedimentation was also carried out to 5 days, but there was no difference in the amount of settling beyond 12 h. As seen in Figure 2c, the 10 and 100 mg/mL suspensions in ethanol that were subsequently used do show degrees of sedimentation over the course of hours, thus prompting vortexing immediately prior to use.

DLS analysis showed that TiO_2_ particles suspended in water had a larger average diameter (225.3 ± 2.6 nm) compared to those in ethanol (221.9 ± 4.7 nm). These values agree with those reported by others who characterized similar suspensions.^15,21^ The particles in both solvents experienced some degree of agglomeration, particularly at higher concentrations, but were generally dispersed similarly in water and ethanol. Less agglomeration was anticipated in water due to its higher polarity, thus yielding an increased zeta potential and greater repulsive force between the TiO_2_ particles.^22^ The similar agglomeration of TiO_2_ in water and ethanol suggests that the solvent effects on agglomeration do not dominate the suspension behavior. Instead, it indicates that agglomeration was primarily driven by interparticle bonds and preagglomeration during manufacturing.^23^ For surface coating applications, it is preferable to have smaller agglomerates to minimize the thickness of the applied coating. A polydispersity index (PDI) was also tested to determine the variability of the particles’ diameters. In ethanol, the average PDI of TiO_2_ was found to be 0.193 ± 0.112, less than that in water (0.217 ± 0.036). The greater uniformity and smaller size nanoparticle clusters in ethanol could translate to a more homogeneous coating on a target piece. In addition, the higher vapor pressure and lower boiling point of ethanol were observed to facilitate more rapid evaporation from treated surfaces after application. This characteristic ensures quicker removal of the solvent, reducing drying time and minimizing potential interference when applying multiple layers of the nanoparticle solution to a surface. These factors contributed to ethanol’s utilization as a solvent for subsequent experiments.

**Figure 3:**
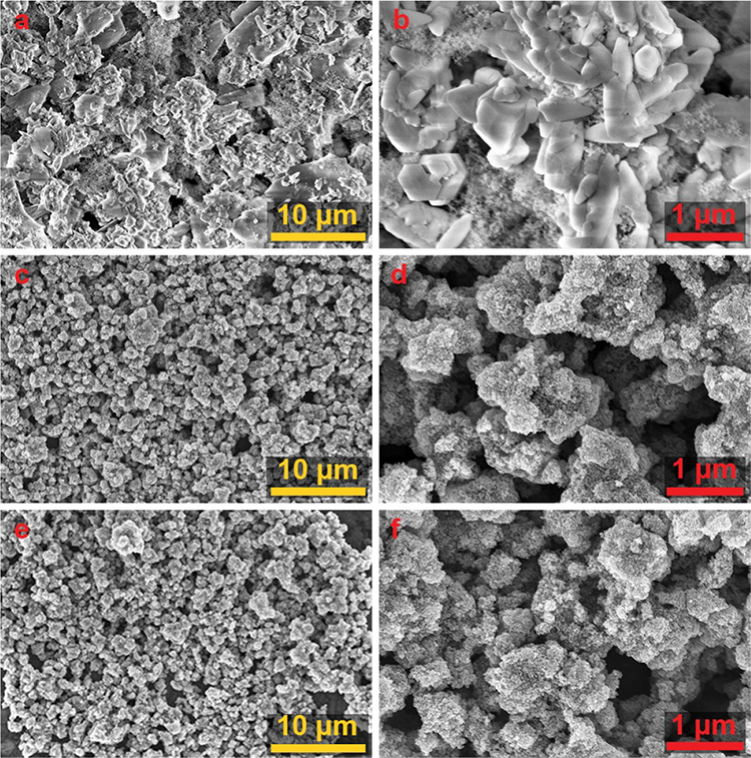
Scanning electron microscopy images indicate differences in surface coverage and the size/shape of the applied coatings. AC-SPS (a, b), 10 mg/mL AB-SPS (c, d), and 100 mg/mL AB-SPS (e, f). Images on the left were acquired at 5000×, with yellow scale bars denoting 10 μm; images on the right were acquired at 40,000×, with red scale bars denoting 1 μm.

Scanning electron microscope imaging further confirmed the presence of agglomeration within the air brush application of solid particle suspensions in solvent (AB-SPS) solutions (Figure 3). The two highest concentrations (100 and 10 mg/mL) both exhibited large agglomerations (Figure 3c,e) that further confirmed the DLS results. At the highest magnifications used, there were no apparent differences in the degree and morphology of agglomerates between the AB-SPS solutions at 10 and 100 mg/mL, suggesting no concentration-dependent agglomeration behavior. Even with some large agglomerations present, individual particles were still visible and were able to evenly coat the surface. The commercially available TiO_2_ spray coating (air canister application of solid particle suspension in solvent (AC-SPS)) produced large crystalline structures that were deposited across the surface along with dispersed TiO_2_ particles (Figure 3a,b). These large crystals are likely from some formulation component used as a propellant or solvent to aid with spraying, similar to previously reported observations of these products.^11,15^

### 3.2 Custom TiO_2_ Surface Coating Enhances Profilometry Resolution to Accurately Evaluate Feature Depth and Coating Roughness

**Figure 4:**
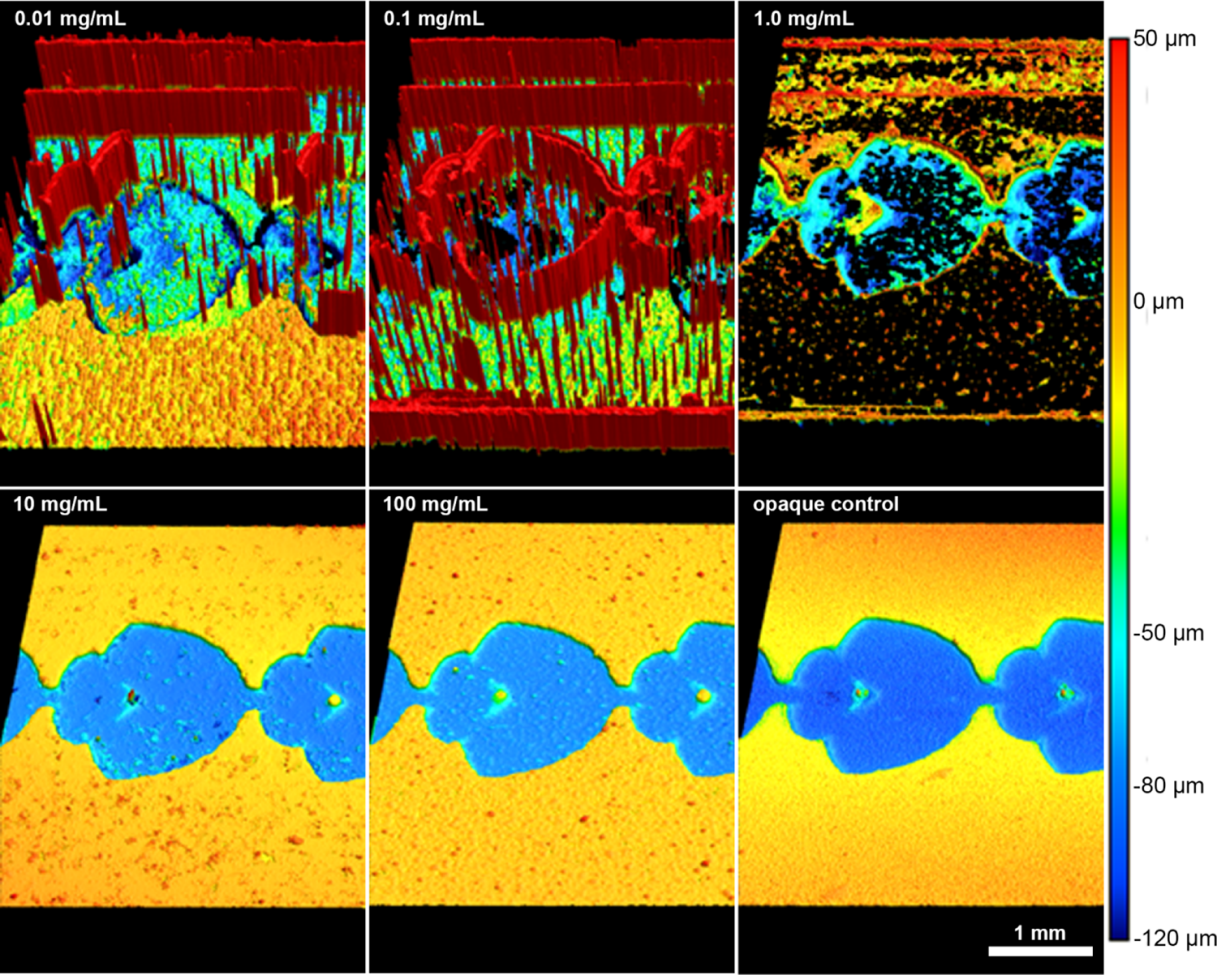
Three-dimensional profilometric scans indicate the scanning performance of different custom TiO_2_ surface coating concentrations when compared to an uncoated opaque control. At lower concentrations of TiO_2_ (0.01 and 0.1 mg/mL), structured light from the profilometer passes through the optically transparent target and onto the profilometer’s baseplate, preventing accurate measurement.

**Figure 5:**
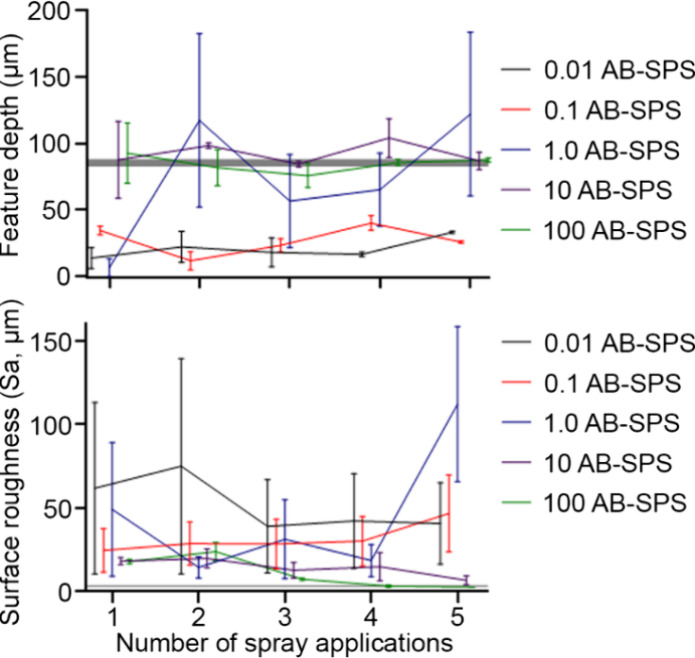
(top) Average scanned feature depth (μm) ± SEM of the microchannel feature on the bottom and (bottom) average surface roughness (μm) ± SEM of a featureless region of the clear part, measured after a varying number of spray applications with AB-SPS treatments. The opaque control, depicted by the horizontal gray line, had a feature depth of 85.3 ± 2.7 μm and a surface roughness of 3.4 ± 0.6 μm.

The various concentrations of the AB-SPS surface coating solution were applied to PDMS substrates, profiled, and analyzed, and the feature depth and surface roughness are presented qualitatively in Figure 4, and quantitatively in Figure 5. The purpose of this test was to determine which concentrations and coating layer counts would be required for accurate scans of the PDMS test piece. Application of the lower AB-SPS concentrations (0.01, 0.1, and 1 mg/mL) resulted in surface roughness measurements that were greater, but not significantly different, than the opaque control. At these conditions, the coating applied was relatively nonuniform, which was reflected with greater variation in measured depths. Such large variance is an indicator that highly variable part geometries were identified in the regions for batchwise analysis and again suggests insufficient coating (Figure 4). Conversely, the higher concentrations (10 and 100 mg/mL) of AB-SPS treatment resulted in lower surface roughness values and accurate feature depth and surface roughness measurements when compared to the opaque control.

Measured feature depths of the microdevice for the 0.01 mg/mL (1 coating layer (*p* < 0.05), 4 (*p* < 0.001), and 5 (*p* < 0.01) layers) and 0.1 mg/mL (1–3 coating layers (*p* < 0.01), 4 (*p* < 0.05), and 5 (*p* < 0.01) layers), were significantly lower than the opaque control. Application of the lowest two concentrations (0.01 and 0.1 mg/mL) resulted in uneven/incomplete surface coating, which made profiling difficult, as so little measurable data were present that subsequent batchwise results became sporadic (Figure 5). At these concentrations, the surface was not effectively coated after 5 layers, leaving an insufficient portion of the PDMS surface rendered reflective, resulting in structured light passing through the microdevice and instead measuring the profiler stage beneath the PDMS piece, thus resulting in much lower feature depths. The 1 mg/mL concentration was more similar to the opaque control at its optimal coating layer (2 coating layers (*p* = 0.9716)), but variance values were large as the stage was being profiled at some locations of inadequate coating coverage. The higher concentration suspensions also showed similar results to the opaque control at their optimal coating layer, 10 mg/mL at 4 layers (*p* > 0.9999) and 100 mg/mL at 5 layers (*p* = 0.9996), and the variances were much smaller than lower concentrations. The application of these higher concentration suspensions effectively eliminated structured light passthrough to the stage, resulting in more accurate profiling. The larger variability of feature depth from the 1 mg/mL concentration likely indicates insufficient coating coverage with 1–5 application layers when compared to the less variable and more accurate results observed at the 10 and 100 mg/mL concentrations.

### 3.3 Low-Cost TiO_2_ Coating Performs Similarly to Commercially Available Alternatives

#### 3.3.1 Comparison of Feature Depth and Surface Roughness on PDMS Casts

**Figure 6:**
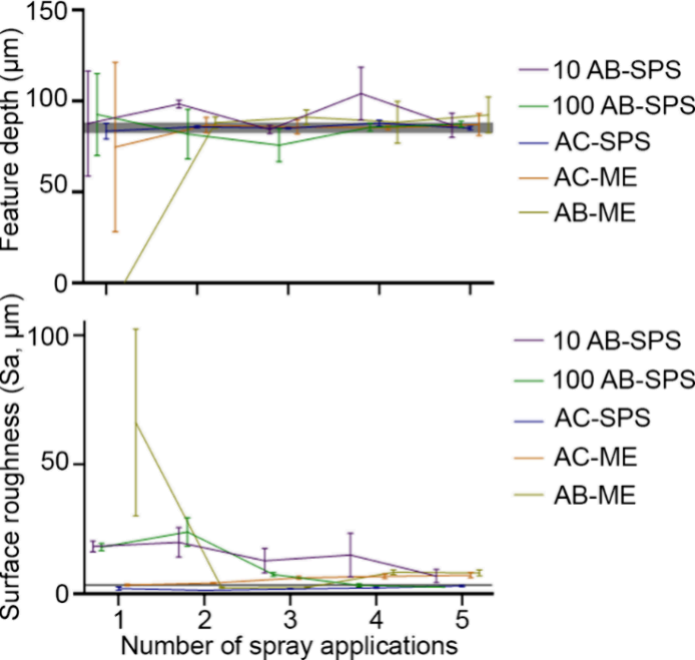
(top) Average feature depth (μm) ± SEM of the microchannel depth and (bottom) average surface roughness (μm) ± SEM of a featureless region of the measuring object, which were measured after a varying number of sprays of AB-SPS, and commercially available treatments were applied. The opaque control, depicted by the horizontal gray line, had a feature depth of 85.3 ± 2.7 μm and a surface roughness of 3.4 ± 0.6 μm.

Feature depth and surface roughness of some common commercially available surface treatments, AC-SPS, AC-ME, and AB-ME (Table 2) were compared to 10 and 100 mg/mL concentrations of AB-SPS. Nearly all of the sprays for each treatment had feature depths that were not significantly different from the opaque control (Figure 6). However, microemulsion (ME) sprays were highly variable after a single coating, with AB-ME being significantly different (*p* < 0.01). After a second coating layer was applied, all surface treatment solutions performed similarly, with the 100 mg/mL concentration of AB-SPS performing the best (4 coating layers, thickness (*p* > 0.9999) and roughness (*p* > 0.9999)). None of the commercial coatings were significantly different from the opaque control.

When selecting the optimal number of layers for a surface coating, it is critical to choose the one that maximizes the feature resolution and coating evenness. This approach differs from the overlap percentage or coverage method commonly found in the literature, where reflective objects are often investigated rather than transparent ones.^11,12,21^ In those cases, full opacification of the surface is essential to minimizing light reflection. While the overlap percentage approach provides insight into the area of the target surface covered by the coating, this measurement is less relevant in this study, as achieving surface coverage does not provide sufficient insight into the quantitative resolution of features on the surface or evenness of the applied coating.

**Figure 7:**
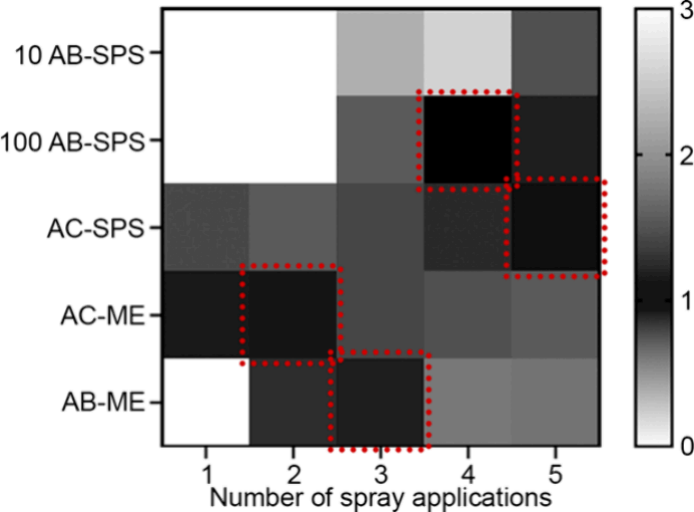
Comparison of the Coating Index values (eq 1.1) of the treatments, and their number of applications, that combine relative depth measurement surface roughness values. Index values of 1 are ideal, with the optimal performance for each treatment indicated by a red dotted box.

To visualize the relative effectiveness of the coatings, the relative accuracy in the measurements of average feature depth and surface roughness to the opaque control were combined in a Coating Index (eq 1.1) and displayed in a heatmap coloration for each surface treatment and its number of spray applications (Figure 7). Values closest to 1 indicated higher performance and were represented by darker colors, while values greater than 3 or near 0 indicated lower performance and were represented by lighter colors. The custom AB-SPS solutions performed better as coating number increased, a trend previously observed in the coverage by similar solutions used.^14^ Similarly, AC-SPS also demonstrated enhanced performance with an increasing number of layers, consistent with a previous report.^11^ In contrast, the commercial solutions AC-ME and AB-ME performed more poorly in higher numbers of coating applications. Interestingly, others observed that, for AC-ME, the optimal overlap percentage was achieved at 6 layers; however, their results also indicated that 2 layers provided similar coverage.^12^ Additionally, 100 AB-SPS performed best at 4 coatings and the lower concentration, 10 AB-SPS, required 5 coating layers, which aligns with the previous findings who reported good coverage with their AB-SPS treatment at 5-layer applications.^21^ AC-ME (1–2 coating layers) and AB-ME (3 coating layers) performed best at lower coatings when compared with 10 AB-SPS, 100 AB-SPS, and AC-SPS.

The optimal number of applied layers was evident for all treatments except AC-ME, where for this treatment, 1 and 2 coating layers had very similar depth measurements (Figure 7) and roughness values (Figure 6). For this treatment, 2 layers were selected as more effective because of its greater reliability in feature depth measurements compared to layer 1 (Figure 6). Moving forward, all treatments were compared when applied at the optimized number of coating layers.

#### 3.3.2 Comparison of Thickness and Roughness for Optimized Spray Coatings

**Figure 8:**
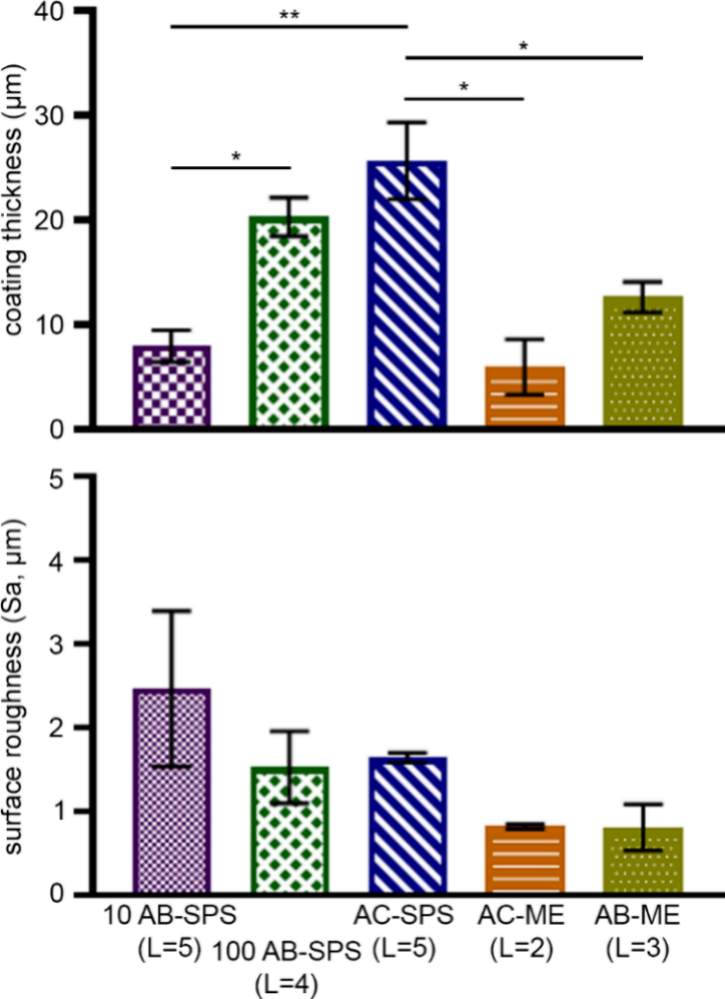
Average thickness of coatings applied to a flat 3D-printed part (μm ± SEM, top) and average intrinsic surface roughness applied to a silicon wafer (μm ± SEM, bottom) of AB-SPS and commercially available treatments, applied at their optimal spray number (as determined from the index values in Figure 7).

To compare the thickness of the coating treatments, a flat 3D-printed piece was coated using the optimal number of surface coating applications from the index described above. As shown in Figure 8, AC-ME and 10 AB-SPS coatings were the thinnest (<10 μm). In contrast, others reported that the AC-ME reached an approximate thickness of 10–20 μm,^12^ which might be due to differences in the movement of the spray nozzle during coating application. Similarly, a coating thickness of AB-SPS treatment was reported to be between 3 and 13 μm,^15^ depending on the application method, which is comparable to the 10 AB-SPS treatment. There was no significant difference between the ME treatments. The 10 AB-SPS was significantly thinner than the other treatments that had a solid particle suspended in a solvent (SPS), (100 AB-SPS (*p* < 0.05) and AC-SPS (*p* < 0.01)), even with its highest number of applied coatings. This discrepancy might have been due to the lower concentration of solid particles in the 10 AB-SPS solution, resulting in a reduced amount of material applied per layer. Considering all treatments, 100 AB-SPS and AC-SPS treatments applied the thickest coatings (>20 μm), with AC-SPS being significantly thicker than 10 AB-SPS (*p* < 0.01), AC-ME (*p* < 0.05), and AB-ME (*p* < 0.05). The thickness of the 10 and 100 mg/mL AB-SPS treatments were significantly greater than the submicron thicknesses reported by Hruboš et al.^21^ However, their coating was specifically tested for coverage on a reflective surface, which suggests that it may not be as effective for applications involving transparent surfaces.

To measure the intrinsic roughness of these coatings, a silicon wafer target object was used that had a surface roughness that was undetectable by the measuring device used in this study (Sa < 0.1 μm). The optimal number of layers for each coating was applied to the target object. As shown in Figure 8, there was no significant difference among any of the evaluated treatments. AC-ME and AB-ME were the only treatments with roughness values below 1 μm, while the 10 AB-SPS treatment was the roughest (>2 μm) and least consistent, as indicated by its large SEM value compared to the others. Considering these results, coatings with higher roughness values may be unsuitable for use on microfluidic devices with feature sizes smaller than the coating’s intrinsic roughness. The custom TiO_2_ solutions were not significantly different from the commercially available options, suggesting promise for their use as an ideal coating for this application, which is even coverage of the clear part to permit accurate feature depth profiling and minimal thickness and roughness so as to not occlude recessed part features and provide consistent readings.

Solvent-surface interactions also play a critical role in predicting the coating performance on different materials, as the polarity of the carrier solvent significantly influences droplet formation during spray application. In the comparison of AB-SPS and commercially available sprays, the SPS sprays were noted to produce more uniform wetting and fewer droplets than the ME sprays when applied to surfaces that are more hydrophilic than PDMS, such as glass (data not shown). Thus, for these part materials, SPS coatings would likely result in a more uniform coating, as the chance of droplets forming is reduced when compared to ME coatings.

#### 3.3.3 Comparison of Costs, Time, and Ease of Use Across Coatings

To further compare the AB-SPS treatment to the commercially available coating options, their cost, time, ease of use, and customizability were considered. As shown in Table 2, the commercial sprays tended to be more expensive than the AB-SPS treatments. However, AC-SPS was less expensive than the 100 mg/mL concentration of AB-SPS but still more expensive than the 10 mg/mL concentration. Outside of the raw costs, the AB-SPS treatment offered more control over the quantity produced as it can be prepared as needed, whereas the commercial sprays required the entire canister or bottle to be purchased. The upfront expense of the air brush system (∼$55 USD) should also be considered.

One advantage of the AC treatments was that they did not require additional hardware cleaning steps following the coating application. The cleaning time required for the airbrush after applying AB treatments made their use more time-consuming compared to AC treatments.^21^ Additionally, the commercial treatments had a faster setup time, since the AB-SPS treatment required preparation and vortexing before application. Furthermore, the SPS treatments took longer to clean from surfaces, as it required the use of an ultrasonicator for complete removal.^15^ ME treatments were noted to be easily removed from surfaces with simple washing.^12^

The AB-SPS treatment offered benefits beyond eliminating single-use aluminum aerosol canisters of the AC treatments. With control over the quantity of nanoparticle suspension that is prepared, the AB-SPS treatment minimized waste compared to commercial sprays, which required full canisters that may not be fully used. Additionally, the AB-SPS treatment involved reusable equipment, such as an airbrush system, which reduced single-use packaging waste. While the use of airbrushes required additional loading and maintenance steps, they operated at fixed pressures, unlike aerosol canisters where pressures may diminish over time, thus potentially altering the coating application. With stable and less harmful chemicals such as TiO_2_ and ethanol, the AB-SPS treatment could potentially lower the environmental footprint, as its composition minimized the likelihood of hazardous or toxic byproducts.^24^ Furthermore, the presence of unknown chemicals in commercial solvents could result in potentially problematic residues on the microfluidic device after cleaning that could compromise the biocompatibility. Finally, although nanoscale TiO_2_ powder has been associated with respiratory hazards and has been classified as a potential carcinogen,^25^ these risks were effectively managed in this study with proper safety measures, such as the use of a standard chemical fume hood when spraying.

The intrinsic properties of the coatings were considered, such as thickness, roughness, and interaction of the carrier liquid with various surface substrates, since these factors can significantly influence the effectiveness of coatings in different applications. For instance, a thinner coating may be favored for microfluidic devices with narrow channel features to prevent feature loss, while low roughness may be advantageous for channels with small z-heights to ensure accurate surface replication. Another important consideration is the selection of a carrier solvent that is compatible with the substrate to achieve a uniform and consistent coating. While the PDMS and resin 3D print substrates tested herein showed no damage following the AC-ME or AC-ME coating applications, it is conceivable that other formulations of 3D resins might not be compatible with the organic solvents or propellants in the microemulsion or aerosol canister coatings.

Using commercial-grade TiO_2_ powder and an inexpensive and portable battery-powered airbrush, this study obtained results that were comparable in many ways to those of studies that employed research-grade TiO_2_ powder and precise spraying tools, such as professional-grade airbrushes or atomizers, which followed tuned application procedures. It should be noted that environmental condition variables such as humidity and temperature could affect the performance of these airbrush-delivered spray coatings, and this study only considered standard laboratory conditions. The intended use of these nanoparticle-based coatings was solely to improve optical profilometry of parts that were otherwise difficult to accurately measure; as such, these coatings were not tested for their durability or longevity. Finally, the versatility of custom TiO_2_-based coating solutions cannot be understated. In the field of rapid prototyping for microfluidics, printed device material composition and transparency can vary. Tunable compositions can allow users to apply appropriate coatings with a minimal impact on surface structures and optimization for measurements of part geometries and surface roughness. In addition, the fully defined chemical composition of these custom coating solutions is preferred in many applications, where unidentified compounds in proprietary commercial treatments could impact the biocompatibility, electrical sensitivity, hydrophobicity, or other device characteristics.

## 4 Conclusions

This study evaluated the effectiveness of commonly used surface coatings in improving the accuracy of surface feature measurements for transparent objects with a focus on microfluidic device features. The effectiveness of a custom TiO_2_ surface treatment in enhancing the measurement of transparent surfaces using optical profilometry was demonstrated and compared to popular commercially available surface coating solutions. The custom TiO_2_-based treatments produced an opaque coating that was uniform enough to replicate the target surface of a transparent PDMS piece. This coating provided the piece with enough resolution for a reliable and accurate measurement of microchannel features across the entire target surface and was found to be as effective as the commercial spray coatings. The commercial treatments (aerosol and microemulsion) were found to be more time-efficient, while the custom TiO_2_ coatings offered better cost-effectiveness, potentially less waste, and customizability. These customizable coatings also allow for a known and controlled composition, providing greater flexibility in surface treatment options.

## Abbreviations

3D: 3-dimension
AB: airbrush
AB-ME: air brush spray application of microemulsion
AB-SPS: air brush application of solid particle suspension in solvent
AC-ME: aerosol canister spray application of microemulsion
AC-SPS: aerosol canister spray application of solid particle suspension in solvent
ANOVA: analysis of variance
CN: China
DLS: dynamic light scattering
FeO: iron oxide powder
FR: France
GRAS: Generally Regarded as Safe
PDI: polydispersity index
PDMS: polydimethylsiloxane
Sa: arithmetical mean height
SEM: scanning electron microscopy
SEM: standard error of the mean
SLA: stereolithography
SLP: structured light profilometry
SPS: solid particle suspended in a solvent
TiO2: titanium dioxide
UK: United Kingdom
USA: United States of America
USD: united states dollars
